# The East African Community (EAC) mobile laboratory networks in Kenya, Burundi, Tanzania, Rwanda, Uganda, and South Sudan—from project implementation to outbreak response against Dengue, Ebola, COVID-19, and epidemic-prone diseases

**DOI:** 10.1186/s12916-021-02028-y

**Published:** 2021-07-09

**Authors:** Muna Affara, Hakim Idris Lagu, Emmanuel Achol, Richard Karamagi, Neema Omari, Grace Ochido, Eric Kezakarayagwa, Francine Kabatesi, Anatole Nkeshimana, Abdi Roba, Millicent Nyakio Ndia, Mamo U. Abudo, Alice Kabanda, Etienne Mpabuka, Emil Ivan Mwikarago, Philip Ezekiel Kutjok, Donald Duku Samson, Lul Lojok Deng, Nyambura Moremi, Maria Ezekiely Kelly, Peter Bernard Mtesigwa Mkama, Alex Magesa, Stephen Karabyo Balinandi, Godfrey Pimundu, Susan Ndidde Nabadda, Dewi Ismajani Puradiredja, Julia Hinzmann, Sophie Duraffour, Martin Gabriel, Gerd Ruge, Wibke Loag, Rogers Ayiko, Stanley Serser Sonoiya, Juergen May, Michael J. Katende, Florian Gehre

**Affiliations:** 1 Department for Infectious Disease Epidemiology, Bernhard-Nocht-Institute for Tropical Medicine, Hamburg, Germany; 2 East African Community (EAC), Arusha, Tanzania; 3 National Institute of Public Health, Ministry of Health and Fight Against AIDS, Bujumbura, Burundi; 4 National Public Health Laboratories, Ministry of Health, Nairobi, Kenya; 5 National Reference Laboratory Division, Rwanda Biomedical Centre, Ministry of Health, Kigali, Rwanda; 6 Public Health Laboratory and National Blood Transfusion Centre, Ministry of Health, Juba, South Sudan; 7 Ministry of Health, Community Development, Gender, Elderly and Children, Dodoma, Tanzania; 8 National Health Laboratory, Quality Assurance and Training Centre, Dar es Salaam, Tanzania; 9 Uganda Virus Research Institute (UVRI), Entebbe, Uganda; 10 National Health Laboratory and Diagnostic Services (NHLDS), Ministry of Health, Kampala, Uganda; 11 Virology Department, Bernhard-Nocht-Institute for Tropical Medicine, Hamburg, Germany; 12 German Center for Infection Research (DZIF), partner site Hamburg–Lübeck–Borstel–Riems, Hamburg, Germany; 13 Tropical Medicine II, University Medical Center Hamburg-Eppendorf (UKE), Hamburg, Germany

**Keywords:** East African Community, Viral haemorrhagic fevers, Ebola virus disease, Dengue fever, Mobile laboratory, COVID-19, Outbreak response, BSL4, Capacity building

## Abstract

**Background:**

East Africa is home to 170 million people and prone to frequent outbreaks of viral haemorrhagic fevers and various bacterial diseases. A major challenge is that epidemics mostly happen in remote areas, where infrastructure for Biosecurity Level (BSL) 3/4 laboratory capacity is not available. As samples have to be transported from the outbreak area to the National Public Health Laboratories (NPHL) in the capitals or even flown to international reference centres, diagnosis is significantly delayed and epidemics emerge.

**Main text:**

The East African Community (EAC), an intergovernmental body of Burundi, Rwanda, Tanzania, Kenya, Uganda, and South Sudan, received 10 million € funding from the German Development Bank (KfW) to establish BSL3/4 capacity in the region. Between 2017 and 2020, the EAC in collaboration with the Bernhard-Nocht-Institute for Tropical Medicine (Germany) and the Partner Countries’ Ministries of Health and their respective NPHLs, established a regional network of nine mobile BSL3/4 laboratories. These rapidly deployable laboratories allowed the region to reduce sample turn-around-time (from days to an average of 8h) at the centre of the outbreak and rapidly respond to epidemics.

In the present article, the approach for implementing such a regional project is outlined and five major aspects (including recommendations) are described: (i) the overall project coordination activities through the EAC Secretariat and the Partner States, (ii) procurement of equipment, (iii) the established laboratory setup and diagnostic panels, (iv) regional training activities and capacity building of various stakeholders and (v) completed and ongoing field missions. The latter includes an EAC/WHO field simulation exercise that was conducted on the border between Tanzania and Kenya in June 2019, the support in molecular diagnosis during the Tanzanian Dengue outbreak in 2019, the participation in the Ugandan National Ebola response activities in Kisoro district along the Uganda/DRC border in Oct/Nov 2019 and the deployments of the laboratories to assist in SARS-CoV-2 diagnostics throughout the region since early 2020.

**Conclusions:**

The established EAC mobile laboratory network allows accurate and timely diagnosis of BSL3/4 pathogens in all East African countries, important for individual patient management and to effectively contain the spread of epidemic-prone diseases.

## Background

The East African Community (EAC) is a regional intergovernmental organisation that consists of six partner states, namely the Republic of Uganda, Republic of Kenya, Republic of Burundi, Republic of Rwanda, Republic of South Sudan and the United Republic of Tanzania. East Africa is home to a population of 170 million people and is prone to frequent infectious disease epidemics. In recent years, these included outbreaks of Marburg virus disease (Uganda-Kenya border; 2017) [1, 2], Rift Valley fever (Uganda, South Sudan; 2018) [3, 4], Crimean-Congo haemorrhagic virus fever (Uganda 2017–2019) [5], Dengue fever (Kenya, Tanzania; 2017/18/19), Chikungunya fever (Kenya; 2016/18) [6], Yellow fever (South Sudan; 2018/20) [7] and Kala Azar/visceral leishmaniasis (Kenya; 2017) [8], to name but a few. Although no recent Zika virus outbreaks were described in East Africa, the virus was originally discovered in a forest in Uganda, 1947, and shortly after also described in Tanzania [9]. Cholera epidemics are frequently occurring in the whole region every year [10]. Most importantly, between 2018 and 2020 (and with a current flare-up in 2021), the Ebola virus disease (EVD) epidemic on the Eastern Democratic Republic of Congo (DRC)/EAC border (the second-largest EVD outbreak in the world) spilled over twice into neighbouring Uganda in 2019 [11]. By June 2020, when the EVD outbreak in DRC was declared over, it claimed the lives of 2287 people. Since 2020, all EAC countries have been affected by the COVID-19 pandemic, with 172,094 confirmed cases and 2523 reported deaths as of February 2021.

The EAC operates by the treaty for the establishment of the EAC, which was signed on November 30, 1999, for partner states “to take joint action towards the prevention and control of communicable and non-communicable diseases and to control pandemics and epidemics of communicable and vector-borne diseases such as HIV-AIDS, cholera, malaria, hepatitis and yellow fever that might endanger the health and welfare of the residents of the Partner States,....”

One of the biggest challenges, and common to all abovementioned outbreaks, is that they are mostly imported into the EAC region via highly frequented, porous borders (such as the remote EAC/DRC border) or occur in distant areas, where the diagnostic capacity to handle infectious pathogens of Biosecurity Level (BSL) 3/4 is not available. In the case of viral haemorrhagic fevers (VHF), where symptom overlap makes clinical diagnosis difficult, molecular diagnosis requires (potentially risky) sample transport from the peripheral health centre to the centralised National Public Health Labs (NPHLs) (often in the countries’ capitals), which delays the time to diagnosis and might not even yield valid diagnostic results due to sample degradation in transit.

Therefore, there is a real need for rapid diagnostics of epidemic-prone BSL3/4 pathogens at the remote outbreak sites in East Africa. As it is not feasible to evenly establish such a highly specialised laboratory infrastructure across the whole EAC region in stationary laboratories, a regional network of very flexible, mobile laboratories, that can rapidly be deployed into the epicentres of outbreaks was identified as the solution.

Working towards this goal, the EAC Secretariat in 2016 signed a 3-year financing agreement (10 million €, 2017–2020) with the Federal Government of Germany, through the German Development Bank (KfW), to support the establishment of the EAC mobile laboratories network. This newly established capacity means that the East African Region can now respond to a wide range of highly infectious VHF outbreaks, and the mobile laboratories have already been deployed in EVD and Dengue fever outbreak missions in 2019, as well as in national responses against the COVID-19 pandemic in 2020 to present.

### Approach

The present paper outlines the strategy that was pursued during the implementation of the project. Following this methodology will allow colleagues who aim to establish similar projects in Africa and other economic blocks globally, to develop a roadmap for implementation. For a successful operationalization of such a network, the following five key areas were identified and shall be considered: (i) transferring ownership to local partners through regional project coordination, (ii) procurement of equipment and consumables involving regional suppliers, (iii) technical aspects of mobile laboratory design, (iv) training of local laboratory operators and (v) field simulation exercises and actual outbreak responses.

#### Regional project coordination through EAC, BNITM and Ministries of Health/National Public Health Laboratories

**Fig. 1:**
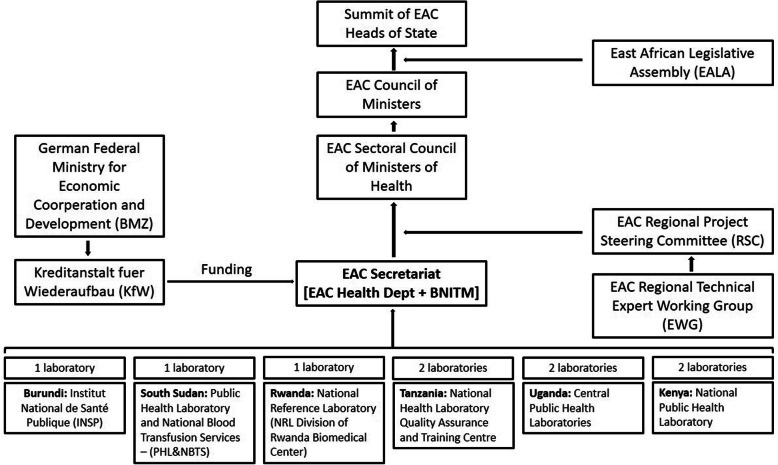
Organogram of project stakeholders and overall integration of the EAC Mobile Laboratories into existing structures

Being the project implementer, the overall project coordination lies with the EAC Secretariat. Given the limited experience in operating mobile BSL3/4 laboratories in most East African countries, the EAC Secretariat (with support from KfW) identified the Bernhard Nocht Institute for Tropical Medicine (BNITM), Hamburg, Germany, a WHO Collaborating Centre for Arbovirus and Haemorrhagic Fever Reference and Research, to provide technical support. In order to effectively advise the EAC Secretariat in the implementation of the mobile laboratory network, the BNITM in 2017 seconded a team of two laboratory consultants (together with administrative support staff) to be permanently stationed at the EAC Secretariat in Arusha, Tanzania (Fig. 1).

On a regional level, the main partners are the respective national Ministries of Health (MoH) and their NPHLs (Fig. 1). By June 2020, the National Public Health Laboratory (Nairobi, Kenya), the Central Public Health Laboratories (Kampala, Uganda) and the National Health Laboratories, Quality Assurance and Training Centre (Dar Es Salaam, Tanzania) each received two mobile laboratories, whereas the Institute National de Santé Publique (Bujumbura, Burundi), the National Reference Laboratory/Division of Rwanda Biomedical Centre (Kigali, Rwanda) and the Public Health Laboratory and National Blood Transfusion Services (Juba, South Sudan) each received one mobile laboratory.

To achieve efficient regional coordination, the EAC is guided by two regional bodies (Fig. 1), both of which convene twice a year.

The expert working group (EWG), a committee of laboratory and emergency response experts from the EAC Partner states and the NPHLs gives essential technical guidance on the establishment and operations of the mobile laboratories. The EWG was actively involved in the conceptualization of the project, design of the laboratories and selection of diagnostic panels and assures that all operational aspects are adjusted to the evolving requirements of the East African region. Most importantly, they facilitate the integration of the laboratories into the respective National Public Health Laboratories diagnostic workflows, national emergency operating centres (EOCs) and respective outbreak task forces.

The Regional Steering Committee (RSC) endorses recommendations made by the EWG. Since the RSC is constituted by representatives of various national ministries, the RSC is crucial in the actual implementation of the laboratories on the highest political level and assures that all project activities are in compliance with national rules and regulations. Besides their outlined essential contributions to the execution of the project, the EWG and RSC routinely guide the EAC Secretariat, who subsequently present ongoing progress reports at the bi-annual EAC Sectoral Council of Health Ministers, whilst also seeking feedback.

The project progress and activities are reported to the EAC Council of Ministers, the East African Legislative Assembly (EALA) and ultimately to the annual EAC Heads of States Summit. Such a structure ensures that the recently established mobile laboratory network is effectively integrated into national outbreak response systems, providing a mechanism for both effective and sustainable use.

#### Procurement of laboratory equipment and consumables

Nine laboratories were procured through a public international tender process. The technical specifications for the mobile laboratory were developed by the BNITM technical experts based at the EAC Secretariat, in conjunction with the laboratory experts in the National Public Health Laboratories in the EAC Partner States. All specifications were approved by the project EWG/RSC, before advertisement by international tender according to KfW/EAC regulations. Laboratory experts and biomedical engineers from each Partner State participated in the technical evaluation of all bids received from the tender, as well as the acceptance of goods delivered to the EAC. One priority was to identify suppliers within the East African region or wider sub-continent, to enable a sustainable mechanism for procurement of equipment and consumables by the NPHLs beyond the duration of this project. In addition, for key diagnostic equipment, suppliers provided onsite technical training for the biomedical engineers, so that they can provide routine preventative maintenance and servicing of the mobile laboratory equipment.

#### EAC mobile laboratory setup

The EAC mobile laboratories are based on the blueprint of the European Mobile Laboratories (EMLab) [12] and comprise a network of nine specialised and portable laboratory units distributed across the East African region through the National Public Health Laboratories, capable of diagnosing infectious pathogens of BSL 3/4. This capacity means that the mobile laboratories can be utilised to respond to a wide range of highly infectious viral haemorrhagic fever outbreaks affecting the East African Region. More recently, the mobile laboratories have been utilised to support SARS-CoV-2 diagnostics throughout East Africa.

The modular nature of the EAC mobile laboratories, which are packed in rugged boxes, means that they are very portable and therefore flexible to the mode of transportation during deployment. The mobile laboratories come with two Land Cruiser vehicles (76 and 78 series, Africa-spec) to transport the equipment and personnel required to operate the laboratory. However, if necessary, the laboratories can be transported by plane, train or ship, to reach remote areas with limited access. This allows for a rapid response to an outbreak, which is important, given that disease confirmation by laboratory diagnosis can be time-critical for effective outbreak containment.

The setup of the mobile laboratory is also flexible due to its modular arrangement. The laboratory can be set up in any existing infrastructure, such as an empty classroom in a vacated school, a community centre or a health facility. Alternatively, it could also be set up in a tent if in a location with no infrastructure. With a power supply system that can either integrate into existing grid power or run from a 3000W generator, with a backup UPS system, the mobile laboratory can operate completely independently. Furthermore, the laboratories are equipped with a military-spec 12V lighting system that can be operated through the electrical system and UPS.

**Fig. 2:**
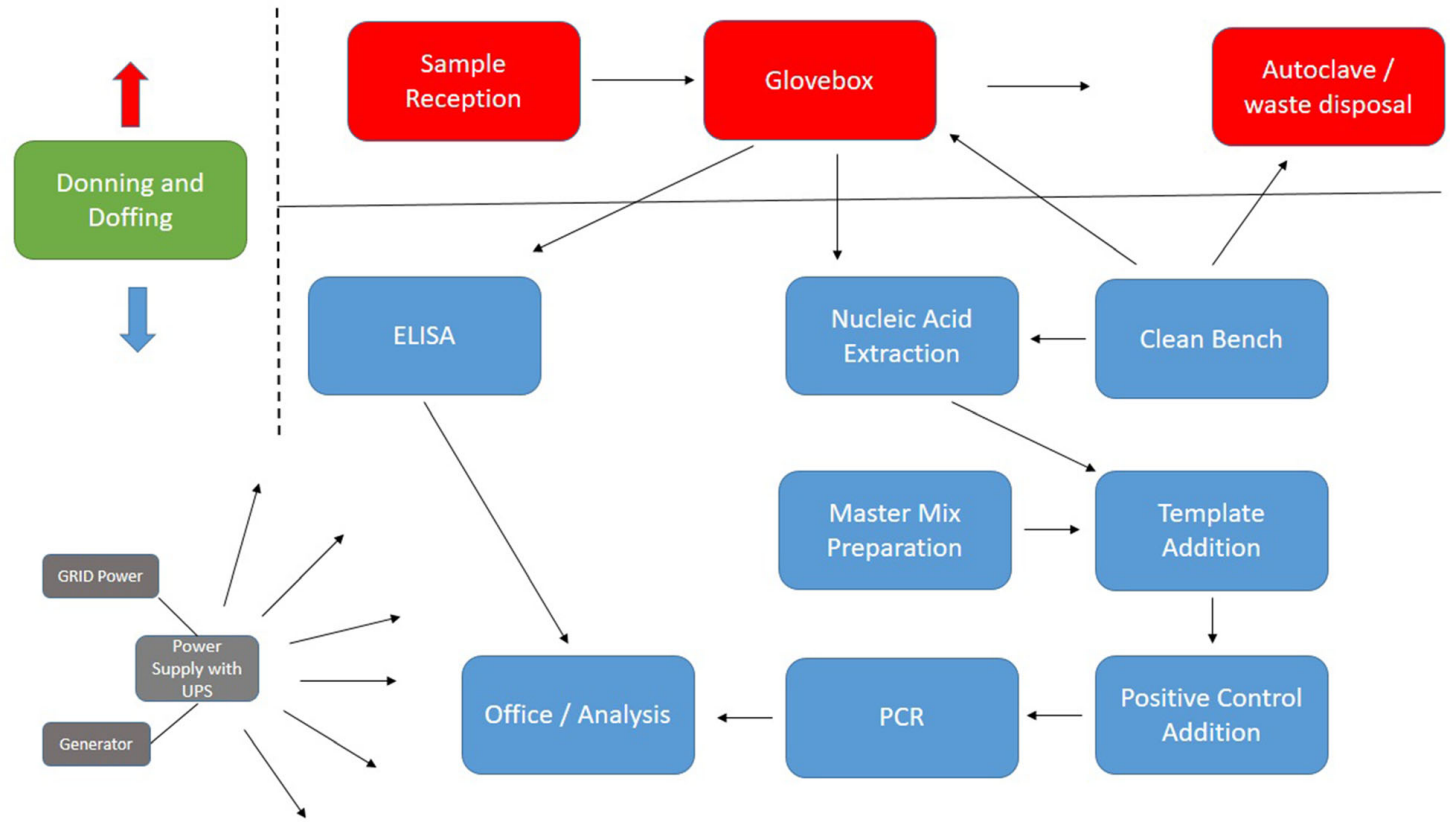
Sample processing workflow of the EAC Mobile Laboratory. A typical workflow in the mobile laboratory with separation of donning and doffing area (green), dirty area (red) and clean area (blue). All mobile lab workstations are connected through the independent power supply (grey), which provides voltage stabilisation to sensitive equipment and UPS backup

The mobile laboratory is designed with a unilateral workflow, in line with its purpose of patient diagnostics. The laboratory can be divided into 12 workstations, as follows: donning and doffing area, sample reception, glovebox for sample inactivation, clean bench for reagent preparation, nucleic acid extraction bench, master mix preparation bench, template addition bench, positive control bench, PCR workstation, ELISA workstation, office area and waste disposal/autoclaving area. For storage of temperature-sensitive diagnostic kits, the laboratories are equipped with two −21°C portable compressor fridges/freezers that can also be powered through the 12V electrical system of the cars and therefore used for transporting kits. To support the cold chain, Va-Q-tainers are provided as well. In general, when selecting equipment, it was considered that machines are mostly maintenance-free, have few moving or otherwise sensitive parts, are resistant to the 0.5% bleach environment of the laboratory, and are generally suited for operating in dusty, hot and humid conditions in East Africa. Although the layout of the laboratory may vary depending on the infrastructure it is being set up in, the general workflow of dirty area (sample reception, glovebox, waste area) and clean area (nucleic acid preparation, PCR, ELISA and office) are maintained. Figure 2 provides an example workflow in the mobile lab. Table 1 explains the activities at each workstation. Ideally, six trained personnel are required to efficiently run the various workstations in the mobile laboratory.

**Table 1:** Activities at each workstation in the mobile laboratory

Work station	Activity
**1.) Donning and Doffing**	Donning and doffing of PPE is always done with a buddy. The level of PPE is dependent on the activity in the lab. Sample reception: Lab coat, nitrile gloves, heavy duty gloves, gum boots, face shield and Tychem apron; Inside the mobile lab: Lab coat and nitrile gloves; Spillage or other exposed situations: Tyvec suit, Tychem apron, nitrile gloves, hood with respirator unit.
**2.) Sample reception**	During reception of infectious samples into the laboratory they are checked for triple packaging and re-packaged under 0.5% bleach if necessary. All packages are decontaminated before entry into the mobile lab using 0.5% bleach. Samples are logged in to the system for onward analysis in the laboratory. A buddy system is used to cross check reception.
**3.) Glovebox**	The glovebox is a containment box under negative pressure, with a Particulate 3 filter and operated using a buddy system, to ensure that all steps of the process are cross checked. Triple packed infectious samples received into the mobile lab are inactivated in the glovebox, either by chemical lysis or heat, depending on the downstream assay.
**4.) Clean bench**	The clean bench area is for reagent preparation. This area contains no samples or extracted nucleic acid material.
**5.) Nucleic acid extraction area**	Extraction of viral RNA/DNA or bacterial DNA for diagnostic PCR.
**6.) Master mix preparation area**	A mobile PCR hood for clean preparation of PCR master mixes. No samples or positive control material enters the hood.
**7.) Template addition area**	Extracted nucleic acid is added to the PCR master mix on a bench area outside of the hood, using a separate set of pipettes.
**8.) Positive control addition area**	The positive control for the PCR reaction is added on a separate bench next to the PCR machine, using a separate set of pipettes.
**9.) PCR workstation**	Bench with CFX96 PCR machine for amplification of viral/bacterial genetic material
**10.) ELISA workstation**	Bench containing the Tecan HydroFlex plate washer and Tecan Infinite absorbance reader
**11.) Office**	A clean area in the mobile lab with a laptop and printer for analysis of results and generation of diagnostic reports.
**12.) Autoclave/waste disposal**	A portable autoclave is provided to inactivate infectious waste.

#### The diagnostic testing capacity of the EAC mobile lab

The mobile laboratories are equipped with BioRad CFX96 PCR machines and Tecan Infinite ELISA absorbance readers, to enable sensitive molecular diagnostics of pathogens causing outbreaks in remote locations. The mobile gloveboxes (Koennecke, Berlin, Germany) of the laboratories provide the capacity to contain and inactivate pathogens of BSL 3 and 4.

During the first phase of the mobile lab implementation, diagnostic pipelines and harmonised standard operating procedures (SOPs) have been established to diagnose a range of viral hemorrhagic, gastrointestinal and respiratory pathogens, as outlined in Table 2. Nucleic acid extraction and diagnostic kits were selected based on compatibility with the CFX96 platform used in the mobile laboratory as well as on independent diagnostic test evaluation by the Foundation of Innovative Diagnostics (FIND) and WHO. In addition, the availability of regional distributors in the East Africa region was taken into consideration to ensure sustainable procurement. Due to the open nature of the diagnostic systems within the mobile laboratory, the repertoire of diagnostic assays is continuously being updated by the EWG/RSC, based on (emerging) priority pathogens affecting the East African Region.

**Table 2:** List of pathogens per testing panel

Viral (Haemorrhagic) fevers	Gastro-Intestinal Panel	Respiratory Panels	ELISA
Crimean-Congo haemorrhagic fever virus	Norovirus GI	SARS-CoV-2	Crimean-Congo haemorrhagic fever virus
Chikungunya virus	Norovirus GII	Influenza A virus (Flu A)	Dengue virus
Dengue virus	Rotavirus	Influenza B virus (Flu B)	Ebola virus
Ebola virus Marburg virus	Adenovirus	Respiratory syncytial virus A (RSV A)	Zika virus
Rift Valley fever virus	Astrovirus	Respiratory syncytial virus B (RSV B)	Lassa fever virus
Zika virus	Sapovirus	Flu A-H1	
West Nile virus (WNV1/WNV2)	*Campylobacter* spp.	Flu A-H1pdm09	
MERS-CoV	*Clostridium difficile* toxin B	Flu A-H3	
Lassa fever virus	*Salmonella* spp.	Adenovirus (AdV)	
Monkeypox virus	*Shigella* spp. / EIEC	Enterovirus (HEV)	
Yellow fever virus	*Vibrio* spp.	Parainfluenza virus 1 (PIV 1)	
	*Yersinia enterocolitica*	Parainfluenza virus 2 (PIV 2)	
	*Aeromonas spp.*	Parainfluenza virus 3 (PIV 3)	
	*Clostridium difficile* hypervirulent	Parainfluenza virus 4 (PIV 4)	
	*E. coli* O157	Metapneumovirus (MPV)	
	STEC (stx1/2)	Bocavirus (HBoV)	
	EPEC (eaeA)	Rhinovirus (HRV)	
	ETEC (It/st)	Coronavirus NL63 (CoV NL63)	
	EAEC (aggR)	Coronavirus 229E (CoV 229E)	
	*Giardia lamblia*	Coronavirus OC43 (CoV OC43)	
	*Entamoeba histolytica*	*Mycoplasma pneumoniae*	
	*Cryptosporidium spp.*	*Chlamydophila pneumoniae*	
	*Blastocystis hominis*	*Legionella pneumophila*	
	*Dientamoeba fragilis*	*Haemophilus influenzae*	
	*Cyclospora cayetanensis*	*Streptococcus pneumoniae*	
		*Bordetella pertussis*	
		*Bordetella parapertussis*	

To assure the reliability of the data generated from the mobile laboratory, a number of internal and external quality control systems have been established within the mobile laboratory platform. Internal quality assurance is carried out in two ways: (1) a subset of samples are sent for independent verification in the central laboratory/reference laboratory in each Partner State and (2) anonymised PCR run files are sent to the scientific expert team located at the EAC Headquarters for independent verification of the results. In addition, all mobile laboratories are enrolled in an external quality assurance (EQA) scheme (Instand, Germany), to enable regular proficiency assessment of the trained personnel working in the mobile laboratory. A laboratory Information Management System (LIMS) is being installed within the mobile laboratories, to enable direct linkage between the mobile labs, the central NPHL and the EAC headquarters. This will enable rapid entry into national databases for outbreak reporting as well as the regional reporting of selected indicators through the EAC.

#### Training activities

**Fig. 3:**
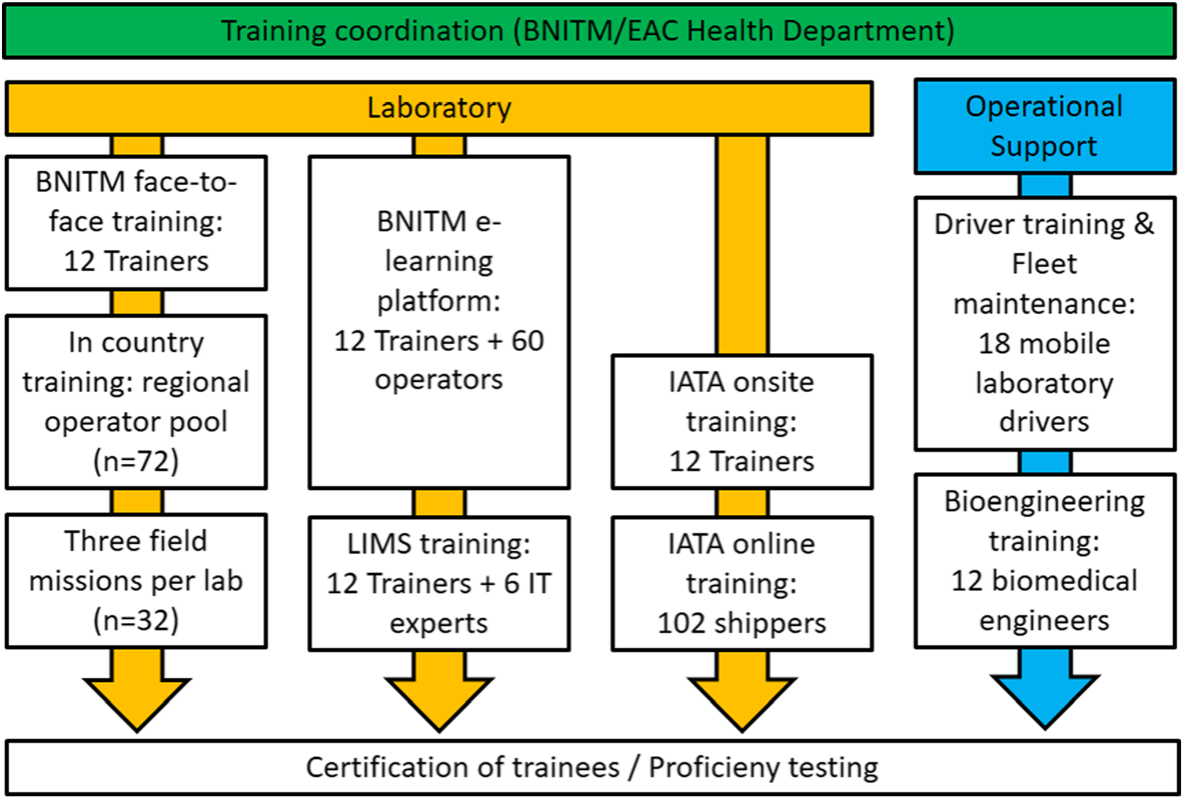
Overview of training coordination and structure

The project is based on a solid training component combining (i) practical laboratory sessions with (ii) an online e-learning platform and (iii) operational support training (see Fig 3).

**Fig. 4:**
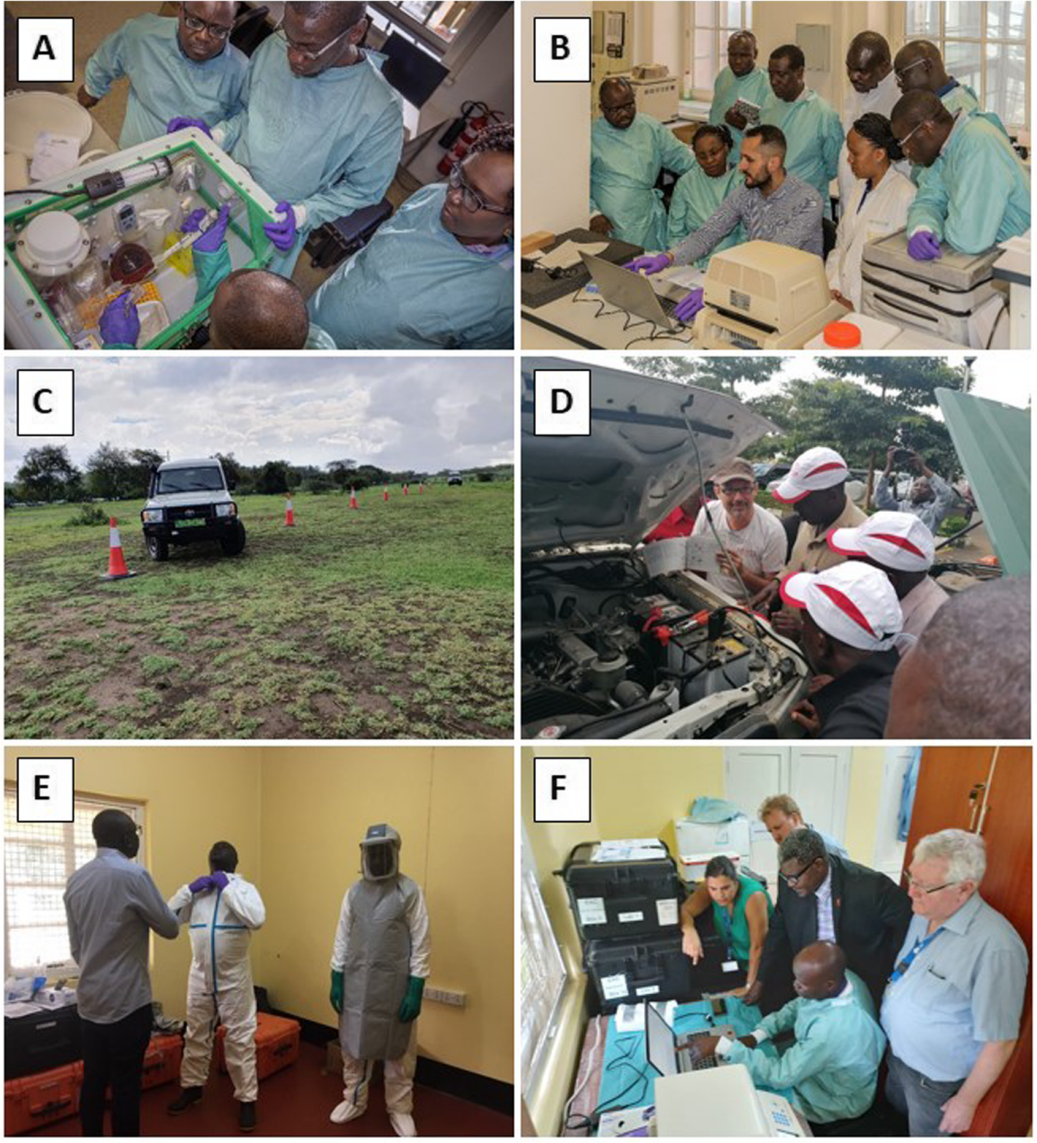
Training activities in the course of the project (2018–2020). **A**, **B** Training in mobile laboratory operations (Oct. 2018) for 12 regional trainers at BNITM, Hamburg, Germany. The training was supported by manufacturers, such as Altona Diagnostics (**B**). **C**, **D** Driver training (Nov. 2019) for 18 drivers from EAC region in safe driving, offroad driving and maintenance of Toyota Land Cruiser 76 and 78 series, in collaboration with Toyota Gibraltar. **E** Ebola Emergency Training (May 2019) in which 12 regional trainers further trained additional operators in the use of the mobile laboratories; here, a South Sudanese regional trainer is educating two additional operators from South Sudan in the correct use of PPE. **F** COVID-19 Emergency training (Feb. 2020): regional trainers were trained in SARS-CoV-2 RT-PCR diagnostics by a team from BNITM

Applying a “Trainer of Trainers” concept, twelve “regional trainers” (2 from each partner state) were trained in-depth for 11 weeks and 6 days in 2018/19 in “mobile laboratory operations, biosafety and diagnostics of BSL3/4 pathogens” (see Table 3). Where appropriate, respective trainings were complemented by experts from the manufacturers/distributors of the equipment (BioRad, Tecan) and diagnostics kits (Altona Diagnostics/Excella, Seegene/Inqaba). In response to emerging EVD outbreaks at the EAC/DRC border in 2019, a Mobile Laboratory Training Facility was established at the EAC Secretariat and several training activities were accelerated to support EVD emergency activities (see Table 3 and Fig. 4). As part of the latter, an on-site IATA training of the regional trainers to facilitator level was conducted in the safe shipping of infectious samples catA and catB, paralleled by online certification of additional 102 shipping staff in the region. The IATA training not only assured that properly triple-packed samples can be transported by air to international reference centres for confirmation of diagnosis, but also that samples referred to the mobile laboratory laboratories were triple packed and potentially risky shipping practices in the region are avoided.

**Table 3:** Overview of all training activities between 2018 and 2021

Training	Content	Date	Location	Duration	Participants	Facilitators
**Introduction to mobile laboratory operations and diagnostic panels**	Operation, BSL3/4 Biosafety and PPE, Workflows, workstations, Diagnostic algorithm and key equipment (Glovebox, CFX96, Tecan ELISA)	October 2018	BNITM, Hamburg	4 weeks	12 regional trainers	EAC/BNITM; Additional user training by BioRad, Tecan, Altona Diagnostics, Seegene/Inqaba
**Ebola virus disease (EVD) emergency response**	Mobile Laboratory Refresher Training	May 2019	EAC HQ, Arusha, Tanzania	1 week	12 regional trainers	EAC/BNITM,
	Accelerated training of additional experts in EVD diagnostics	May 2019	EAC HQ, Arusha, Tanzania	2 weeks	12 regional trainers + 12 trainees	Conducted by regional trainers under BNITM supervision. Excella/Altona Diagnostics
	Proficiency assessment of regional trainers	August 2019	EAC HQ, Arusha, Tanzania	2 weeks	12 regional trainers	EAC/BNITM, Individual assessment of ToTs by independent team.
	IATA training of catA/catB infectious agents and dangerous goods	August 2019	EAC HQ, Arusha, Tanzania	3 days	12 regional trainers (facilitator), 102 shippers (online training)	EAC/BNITM, Transport Development Group (TDG)
	Laboratory Leadership Training	August 2019	EAC HQ, Arusha, Tanzania	3 days	12 regional trainers	EAC/BNITM
**Driver training**	Safety, Maintenance and driver training for operation of Toyota Land Cruiser fleet	November 2019	EAC HQ, Arusha, Tanzania	1 week	18 drivers	EAC/BNITM, Toyota Gibraltar
**COVID-19 emergency response**	SARS-CoV-2 diagnostics	February 2020	EAC HQ, Arusha, Tanzania	2 weeks	6 regional trainers	EAC/BNITM
**Bioengineering training**	Maintenance of mobile laboratory equipment	Scheduled 2021	TBD	3 weeks	12 biomedical engineers	EAC/BNITM/TBD
**LIMS training**	Programmer and user training for the mobile laboratory LIMS	Scheduled 2021	TBD	1 week	12 regional trainers, 6 IT experts	EAC/BNITM

By August 2019, the regional trainers underwent individual proficiency testing, in which they demonstrated their capability to operate the mobile laboratories and process blinded panels from sample reception to releasing results. Of the twelve regional trainers, six were immediately fully proficient at all workstations, 4 more were fully proficient after revisiting details of certain SOPs and 2 regional trainers were certified to only work at certain workstations. By Sept 2019, a deployable regional EAC rapid mobile laboratory response team of 12 operators was available with the limitation that two team members were only allowed to operate at select work stations for which they demonstrated proficiency in the previous individual assessment. In February 2020, and before the COVID-19 epidemic was declared a global pandemic, a SARS-CoV-2 diagnostic training was conducted as part of the EAC’s COVID-19 emergency response.

By June/July 2020, the 12 regional trainers were educating an additional 10 operators in each of the six countries (“in country trainings”), resulting in a regional pool of 72 mobile laboratory operators. Taught knowledge is being reinforced during field simulation exercises and field missions (see Fig. 4), which will continue throughout 2021.

To assure operational support, 18 drivers were trained (in collaboration with Toyota Gibraltar) in the maintenance of the provided Toyota Land Cruiser fleet and safe driving and loading of the trucks. For long-term sustainability, an additional 3-week training of biomedical engineers to support the maintenance of mobile laboratory equipment and a 1-week LIMS training is scheduled for 2021.

All online trainings (included in the e-learning platform) were provided by the German Federal Foreign Office-funded German Online Platform for Biosecurity and Biosafety (GO4BSB). A list of all interactive self-study e-learning modules used by the EAC mobile labs for their online trainings can be found below (see Table 4). The regional trainers worked through a total of 17 modules, which provide an introduction to biosecurity, laboratory biosafety, epidemiology, good clinical practice and related topics. The learning progress was assessed by means of online pre- and post-tests and the regional trainers’ performance increased over the course of the training programme. During their final proficiency evaluation (Aug 2019) all regional trainers answered more than 90% of test questions correctly. Currently, the additional 60 trainees (educated during the “in country trainings”) are enrolled in the online training.

**Table 4:** Modules included in the online training

No.	Module title
1.	Infectious disease pathogens – general virus biology *Pathogènes infectieux – Biologie générale des virus et d’Ebola*
2.	Diagnostics – DNA/RNA *Diagnostic – ADN et ARN*
3.	Diagnostics – PCR/RT-PCR *Diagnostic – PCR et RT-PCR*
4.	Laboratory biosafety *Biosécurité*
5.	Laboratory biosafety: Donning and Doffing *'équipement de protection individuelle’ (EPI)*
6.	Introduction to biosecurity
7.	International health regulations 2005
8.	Dual-use
9.	Packaging and shipping
10	Public health surveillance
11.	Risk assessment
12.	Disaster response and rapid health assessment
13.	Virus biology and SARS
14.	Antimicrobial resistance Pt. 1 & Pt. 2
15.	Good clinical practice E6 (R2)
16.	Introduction to Good Clinical Laboratory Practice (GCLP)
17.	GCLP: organisation and personnel

The online training was provided by GO4BSB, which is a project of the German Biosecurity Programme and a collaborative initiative by BNITM, Bundeswehr Institute for Microbiology (IMB), Friedrich Loeffler Institute (FLI) and Robert Koch Institute (RKI). GO4BSB is coordinated and hosted by BNITM. Modules 1–5 have been originally developed for the EBOLearn curriculum. These modules are available in English and French. Modules 6–13 have been originally developed for the German Federal Foreign Office funded Global Partnership Initiated Biosecurity Academia for Controlling Health Threats (GIBACHT) fellowship programme (www.gibacht.org). The online training was further supplemented with links to the open access online modules on antimicrobial resistance provided by the Global Health Learning Center (www.globalhealthlearning.org) and the modules on Good Clinical Practice provided by the Global Health Network’s Training Centre (www.globalhealthtrainingcentre.tghn.org)

### Impact

#### Reduction of sample turn-around-time for diagnosis at outbreak sites

The major impact of the project was that countries and the region as a whole are now sustainably equipped with de-centralised BSL-4 infrastructure that can be rapidly deployed to remote areas when outbreaks happen—before the project, similar mobile laboratories had to be deployed from Europe or elsewhere. When no mobile laboratory support was available, samples needed to be shipped for diagnosis from peripheral health centres at the sites of outbreaks to central NPHLs in the capital cities, to regional reference centres (such as the UVRI, Uganda) or to international collaborators in Europe/USA. Therefore, countries previously reported average turn-around-times from sample collection to diagnosis of 30h (Uganda), 48h (Tanzania), 72h (Kenya, Rwanda) and 168h (South Sudan). With the arrival of the EAC mobile laboratories and the training of regional operators, sample turn-around-time at the site of the outbreak was reduced to 8h in all six countries. This has an immediate impact on the time individual patients have to spend in isolation wards (for VHF) and also will interrupt chains of transmission rapidly.

#### Field simulation exercises

**Fig. 5:**
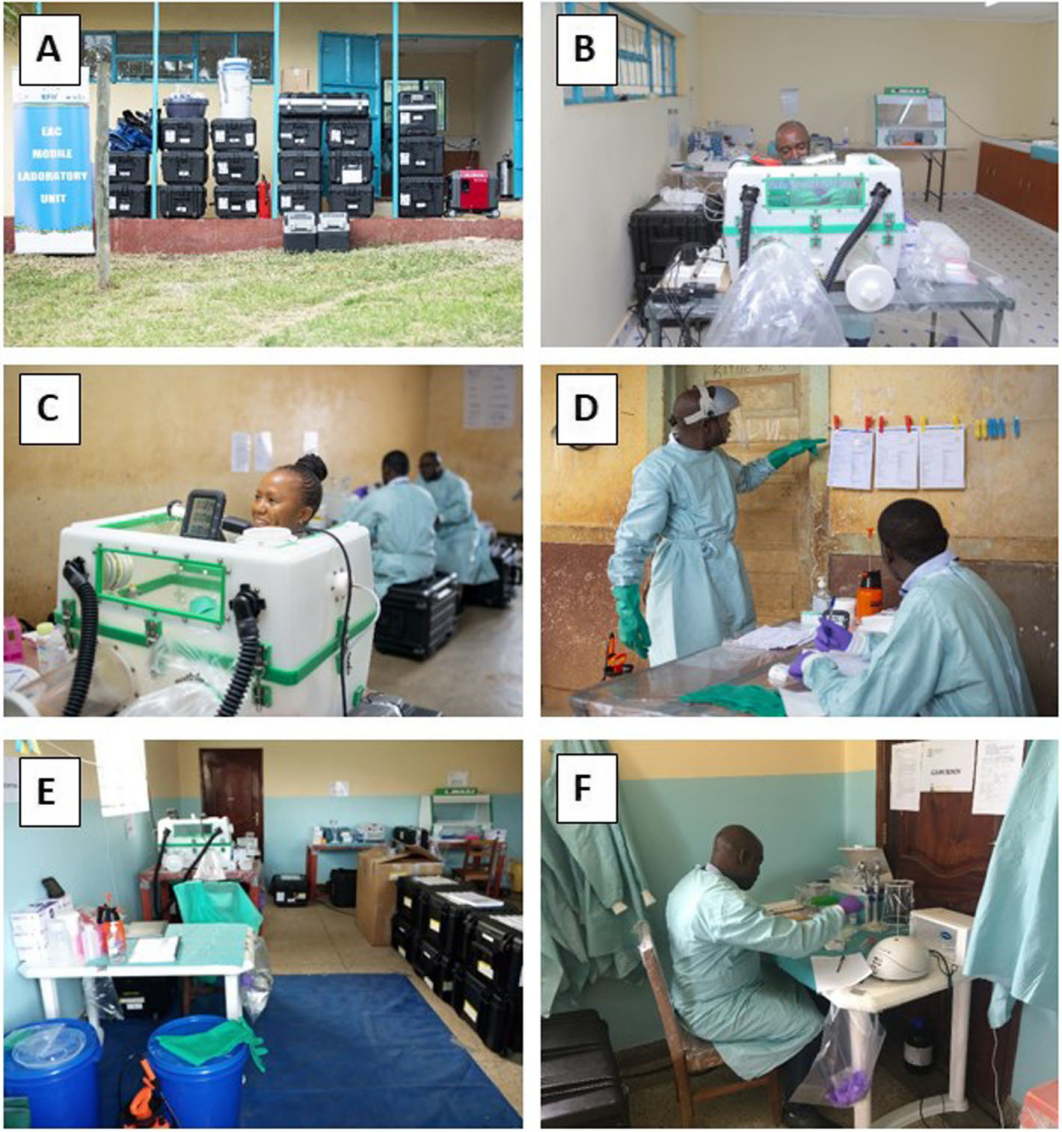
Field simulation exercises and outbreak deployments of the mobile laboratories 2019–2020. **A**–**D** Participation of two mobile laboratories in the EAC field simulation exercise on the Kenya/Tanzania border in Namanga, June 2019. Setup of one mobile laboratory at the Kajiado Regional Health Centre, Kenya (**A**, **B**). Setup of the second laboratory in the Namanga primary school (during school holidays), next to the Namanga Health Centre and Dispensary, Tanzania (**C**, **D**). **E**, **F** Deployment of one laboratory to Kisoro Health Centre, as part of the EAC Ebola Emergency Response, Oct/Nov 2019. The laboratory contributed to the Ugandan preparedness activities and analysed samples collected from the DRC/Uganda border region

To test the successful integration of the mobile laboratories into national emergency outbreak response mechanisms, as well as test the functionality of the lab under field conditions, the EAC mobile laboratories have been deployed during an EAC/GIZ/WHO field simulation exercise that took place at the Namanga border between Kenya and Tanzania in June 2019 [13, 14] (see Table 5). One mobile laboratory was set up on either side of the border, each operated by a team of 6 regional trainers. The deployment allowed the mobile lab teams to assess multiple aspects of operations, not possible to test in the classical classroom settings. These included the packing and transport of the laboratories, ease of border crossing with the laboratory and setting up the laboratory in different infrastructures (in a school classroom on the Tanzanian side and a health facility on the Kenyan side). One major finding was that sample referral from the local health centres to the mobile laboratories needs to be assured in future outbreak missions (Fig. 5A–D).

**Table 5:** Overview of all field simulation exercises and outbreak responses between 2018–2021

Activity	Country	Start Date	Number laboratories	Deployment	Duration
**EAC/WHO Simulation Exercises**	Tanzania/Kenya border (Namanga One Stop Border Post)	June 2019	1	Kajiado Health Centre/Kenya	1 week
			1	Namanga Primary School/Tanzania	1 week
	South Sudan/Uganda border	Scheduled 2021	1	South Sudan	1 week
			1	Uganda border	1 week
**Dengue Fever Support**	Tanzania	August 2019	1	EAC HQ, Arusha	1 week
**Ebola Virus Disease (EVD) Emergency Response**	Uganda	Oct/Nov 2019	1	Kisoro, Uganda/DRC/Rwanda border	6 weeks
**COVID-19 Emergency Response**	Tanzania	June 2020	2	Mabibo	ongoing
	Kenya	June 2020	1	Naivasha, Kenya/Uganda border	ongoing
		June 2020	1	Namanga, Kenya/Tanzania border	ongoing
	South Sudan	June 2020	1	Nimule, South Sudan/Uganda border	ongoing
	Uganda	May 2020	1	CPHL, support of central NPHL	6 weeks
		June 2020	1	Tororo, Uganda/Kenya border	ongoing
			1	Adjumani, Uganda/South Sudan border	ongoing
	Burundi	August 2020	1	Kobero, Burundi/Tanzania border	10 weeks
	Rwanda	June 2020	1	Kirehe, Rwanda/Tanzania border	32 weeks
		January 2021	1	Kigali Int‘l Airport	ongoing

For 2021, another field simulation exercise is scheduled on the border of South Sudan and Uganda, in which two mobile laboratories will be included.

#### Field missions

Since the completion of trainings and the field simulation training exercise in 2019, the mobile laboratories have participated in eleven emergency outbreak missions (one Dengue fever mission, one Ebola virus disease mission, and nine COVID-19 missions) all over East Africa (see Table 5 for an overview). A twelfth mission is currently under planning to respond to a request by the South Sudanese Ministry of Health for diagnostic support to an outbreak of an unknown haemorrhagic fever in the South-West of the country. Tanzania is also currently preparing a thirteenth mission to send one mobile laboratory to Zanzibar to support the SARS-CoV-2 diagnostics on the island.

In 2019, Tanzania experienced an outbreak of Dengue fever in the Tanga region and the capital Dar Es Salaam [15]. Whilst stationed at the EAC, Arusha, the mobile laboratory regional team assisted in the molecular conformation and serotyping of previously collected, rapid diagnostic test-positive, suspected Dengue fever serum samples. In total, 459 suspected serum samples were processed using RealStar Dengue 2.0 kits (Altona Diagnostics) and a subset of 230 positives were serotyped using the RealStar Dengue Type kit (Altona Diagnostics). The major finding was that the 2019 outbreak was caused by a circulating Dengue virus serotype I (manuscript in preparation).

In another mission to respond to the EVD outbreak in Eastern DRC (2018–2020), and the increased risk of spill-over into the neighbouring EAC region [16], in Sept 2019, the Ministry of Health, Uganda, requested a mobile laboratory to support the countries’ EVD preparedness. In Oct 2019, a mobile laboratory was deployed to Kisoro Health Centre serving the DRC/Rwanda/Uganda border region to test blood samples of patients suspected of EVD (Fig. 5E, F). The Ugandan laboratory team was supported by members of the regional, rapid response team deployed from Burundi, South Sudan and Kenya. Using the RealStar FiloScreen 1.0 kit (Altona Diagnostics), twenty-five referred suspect samples were tested and no EVD case was found during the mission (manuscript in preparation).

In March 2020, the first cases of COVID-19 were reported in the EAC region. The six EAC Partner States implemented varying public health measures and all border crossings between countries were temporarily closed. Five countries (Uganda, Kenya, Rwanda, Burundi and South Sudan) deployed seven laboratories to the border regions in order to fast-track SARS-CoV-2 testing at the points of entry and assure smooth cross-border traffic. In Tanzania, SARS-CoV-2 testing was centralised and the National Public Health Laboratory in Mabibo was commissioned to lead the diagnostic response. Both mobile laboratories were utilised to support the NPHL. So far, the EAC mobile laboratory network has screened over 280,000 samples for SARS-CoV-2 in the region.

The project also provided infrastructural support for the centralised NPHLs. For instance, and as part of the regional EVD emergency response, each of the six EAC countries received an additional glovebox and PPE. This was to assure adequate BSL3/4 levels for processing of EVD suspected samples at the central public health laboratories. In addition, and as measures taken during the regional COVID-19 response, the six National Public Health laboratories were equipped with one additional CFX96 PCR cycler each, as well as additional centrifuges to support the high sample throughput in the mobile laboratories. In addition, the laboratories were provided with RNA extraction and diagnostic SARS-CoV-2 kits.

### Recommendations

In order for colleagues to implement similar large-scale regional projects and to establish lasting structures (which remains a key priority for the mobile laboratory project), we recommend to continuously consider aspects of sustainability during project conceptualization, procurement, training development and when concluding on the mandate of the laboratories.

The crucial aspect for project success during conceptualization was that through the overarching coordination of the EAC, the mobile laboratory project has been integrated into the national Ministries of Health and guided by priority gaps identified in the East African region. As such, and in contrast to other mobile laboratory endeavours, the EAC mobile laboratories are an extension of the existing national laboratory infrastructure and are therefore able to be rapidly mobilised based on national decision-making. In addition, as with the central NPHL, operations of the mobile laboratory can be factored into the annual budget allocations for disease outbreak response.

Since public funds were utilised, a competitive, international tender was needed. If other projects have the same obligation and depending on the size of the tender, a full-time procurement expert is recommended, who needs to be familiar with both the funder’s and donor countries’ rules and regulations, and can advise on the most suitable Incoterms for goods delivery. Working closely during project implementation with the governments helped to efficiently apply for customs import clearances, approvals by National Medical Regulatory Bodies (e.g. TMDA in Tanzania), and to transfer laboratories across borders. The duration of such a tender process also needs to be realistically communicated to all stakeholders from the very start of the project. It is recommended to advertise these tenders regionally as well so that local providers and distributors will be able to apply. As local agents are familiar with import regulations and distribution of kits, long-term sustainability of supply chains and equipment servicing will be guaranteed. One important aspect to consider is that export control regulations for dual-use items from exporting countries might apply (e.g. for certain diagnostic kits, PPE, BSL3/4 equipment, etc.) and approvals can take a considerable amount of time to be granted.

Furthermore, a wider and inclusive training strategy was very beneficial during project implementation. Besides training technical laboratory staff (as outlined above), we recommend professional development of administrative staff, IT experts, biomedical engineers and drivers. This not only provides a general sense of ownership by a wider group of people, but also a more effective integration of the project within the NPHL. Such a training strategy will also contribute to the longer term sustainability of the mobile laboratories, with the biomedical engineers, IT experts and drivers with greater capacity to resolve technical issues internally and ensure regular maintenance of the laboratory equipment, vehicles and communication systems.

It was also recognised that one important aspect to ensure sustainability was the frequent use of the mobile laboratory, both to maintain and expand the pool of trained personnel and to enable the mobile laboratory to be fully integrated into the national systems. Given that disease outbreaks can be sporadic, there was a risk that the laboratories would be packed away and underutilised. It was therefore decided to expand the mandate, so that the mobile laboratories also support national disease surveillance activities. As such, the mobile laboratories can improve accessibility to samples from remote areas, where transport to the central laboratory often results in poor viability. In addition, the mobile laboratories can also be utilised as a research platform, providing collaborators with unparalleled access to conduct research in the six EAC Partner States. Through funding personnel and the operation of the mobile laboratory under deployment, the research platform could form a sustainable funding stream to maintain the mobile laboratories.

## Discussion

From 2017 to 2020, a network of nine mobile laboratories across six countries was established in the EAC region that allows a de-centralised response to BSL-4 pathogens in East Africa. For the project to work independently and sustainably, it was important to transfer project ownership to the regional coordinators and stakeholders from the very beginning. Since the laboratories will have their greatest benefit providing diagnostic services in outbreaks, NPHLs were considered to be the optimal recipients (rather than research institutions). With the EAC being the implementer and by the inclusion of an expert working group (guiding all decisions), as well as an actively involved regional steering committee, political buy-in on the highest level was guaranteed. In order to rapidly respond to changing circumstances, frequent, bi-annual EWG/RSC meetings are essential. During these meetings, joint decisions are made and reports are signed off based on consensus. The mobile laboratories were eventually handed over to the Ministries of Health in 2020 and can thus be independently coordinated through the NPHL and operated by the trained national teams. Therefore, project ownership was completely transferred to the stakeholders in the region, reducing the need for outside assistance to a minimum. Consequently, the laboratories were already utilised in one field simulation exercise and eleven outbreak missions, during which certain challenges, lesson learnt and limitations were observed.

During the field simulation exercise, it became apparent that efficient referral of suspect samples from health centres to the mobile laboratory can be challenging. To avoid a lack of samples in real-life outbreak missions, the respective MoHs/NPHLs assured that the mobile laboratories will be represented in the National Task Forces and Emergency Operating Centres (EOC) and therefore fully integrated in country-wide epidemic responses, resulting in a rapid deployment into outbreaks, access to national funding and referral of suspect samples.

Limitations to still overcome are that importation and clearance of donated laboratory equipment and diagnostic kits remain difficult in some countries and that countries are, to a certain degree, relying on outside support for diagnostic kits. This became especially apparent during the COVID-19 pandemic, in which countries were facing much higher sample volumes, beyond the originally anticipated capacity of the mobile laboratories. Although the project increased the throughput capacity by adding PCR machines and centrifuges, countries still relied on receiving donations of nucleic acid extraction and SARS-CoV-2 diagnostic kits (from various manufacturers), potentially interrupting established workflows through harmonised SOPs in the mobile laboratories. Given that several months of continuous field missions (as seen during the COVID-19 responses) is not only expensive, but also a burden on the deployed laboratory staff, some countries decided to train further laboratory staff resident of the deployment areas, in mobile laboratory operations, thus further expanding decentralised capacity.

## Conclusion

Although fully operational, the newly established EAC mobile laboratory network needs to achieve the next critical steps for full integration into the host institutions.

Over the next years, the network will be linked with existing quality systems in the NPHLs, so that they can seek accreditation through official bodies, of which many of the participating central laboratories already have. It is also important that this regional laboratory network connects with other organisations in the African continent, such as CDC Africa, WHO AFRO and ASLM, to synergise efforts in supporting partner states to respond to disease outbreaks.

The second big task is to extend the mandate beyond VHF. One priority area identified was surveillance of the Anti-Microbial Resistance (AMR) surveillance in the East African region that could not be fully addressed with the existing mobile laboratory infrastructure. Such bacterial diagnosis and resistance profiling are not possible in the existing mobile laboratory (which lacks bacterial culture facilities). The mobile laboratory network will therefore be enhanced through the addition of mobile BSL3 container laboratories, equipped with bacterial culture facilities and the capacity to conduct whole-genome sequencing (2021–2024). The container laboratories will both support the existing rapidly deployable mobile laboratories with enhanced technologies, as well as enable routine AMR surveillance to be carried out in the East African Region.

## Data Availability

Not applicable

## Abbreviations

EAC HQ: East African Community Headquarters
BNITM: Bernhard-Nocht-Institute for Tropical Medicine
UVRI: Uganda Virus Research Institute
NPHL: National Public Health Laboratory
KfW: Kreditanstalt für Wiederaufbau
DRC: Democratic Republic of Congo
BSL: Biosafety level
VHF: Viral haemorrhagic fever
EVD: Ebola virus disease
MoH: Ministry of Health
EWG: Expert Working Group
EOC: Emergency Operating Centre
RSC: Regional Steering Committee
EALA: East African Legislative Assembly
PCR: Polymerase chain reaction
ELISA: Enzyme-linked immunosorbent assay
FIND: Foundation for Innovative New Diagnostics
LIMS: Laboratory Information Management System
IATA: International Aviation Transport Agency
GO4BSB: German Online Platform for Biosecurity and Biosafety
Fsx: Field simulation exercise
RDT: Rapid diagnostic test
TMDA: Tanzanian Medical Devices Agency
